# Tracheo-Laryngotomy

**Published:** 1891-04

**Authors:** D. B. McGehee

**Affiliations:** Brownwood


For Daniel's Texas Medical Journal.

### TRACHEO-LARYNGOTOMY.

BY D. B. M'GEHEE, M. D., BROWNWOOD.

IF YOU will kindly lend me space in your valuable journal, I will give you the notes on a case of foreign body in air passages, and operation for same.

Case: Iler A., female, age, 6 years, healthy robust child, while playing with some other children, July 6, 1890, was supposed to have sucked one, of some No. 3, buckshot into her trachea; but on examination I concluded it was a "false alarm," and returned, directing to be summoned if any symptoms developed. Heard nothing more from the little patient till July the 9th, when I was called in great haste. This time physical examination revealed total absence of respiratory murmer on right side, but no dullness; there was slight cough with expectoration of some little blood, no pain. I took the little fellow up in my arms, with the body inverted, and attempted to make her expel the offending body by force of gravity. Upon auscultation, I found the foreign body had changed sides; and was now in left bronchus, producing some physical signs as before, only on opposite sides. I then informed the parents that an operation was imperatively demanded, to which they readily consented. So next morning (July 10th,) I in company with my friend Dr. Y., repaired to the house of our little patient and did a tracheo-laryngotomy, by which we succeeded in removing a No. 3 buckshot, after some little difficulties, owing to its leaden nature. I do not mention this because of any peculiarity attached to the operation, but of the rarity of leaden missiles getting into the air passages, and the liability of some unwary fellow to mistake it for pneumonia, as an M. D. (!) was passing this gentleman's house the day before we operated, and diagnosed pneumonia, warning the family against allowing an operation.

